# Skeletal Stability Following LeFort I Maxillary Advancement With Rigid Fixation: A Retrospective Study

**DOI:** 10.7759/cureus.99341

**Published:** 2025-12-15

**Authors:** Sunil Kumar Gulia, Lalitha Singu, Jhansi Rani, Rajkumar Patel, Samir Mansuri, Rahul VC Tiwari, Heena Dixit, Seema Gupta

**Affiliations:** 1 Department of Oral and Maxillofacial Surgery, Shree Guru Gobind Singh Tricentenary University, Gurugram, IND; 2 Department of Dentistry, Complete Dental of Suwanee, Suwanee, USA; 3 Department of Oral and Maxillofacial Surgery, Mamata Institute of Dental Sciences, Hyderabad, IND; 4 Department of Dentistry, Boston University Henry M. Goldman School of Dental Medicine, Boston, USA; 5 Department of Oral and Maxillofacial Surgery, Al Kuwait Hospital, Dubai, ARE; 6 Department of Oral and Maxillofacial Surgery, Ram Krishna Dharmarth Foundation (RKDF) Dental College and Research Centre, Bhopal, IND; 7 Department of Public Health, Commissionerate of Health and Family Welfare, Hyderabad, IND; 8 Department of Orthodontics, Kothiwal Dental College and Research Centre, Moradabad, IND

**Keywords:** advancement, lefort i, maxillary, orthognathic, osteotomy, relapse

## Abstract

Introduction

Postoperative skeletal relapse is the most common complication following LeFort I maxillary advancement, even with modern rigid fixation techniques. This retrospective study aimed to evaluate the magnitude and predictors of horizontal skeletal relapse at one year after Le Fort 1 advancement osteotomy stabilized exclusively with rigid fixation.

Materials and methods

Thirty-two consecutive adult patients (mean age, 25 years) who underwent LeFort I advancement (with or without simultaneous mandibular surgery) between 2018 and 2021 were included after obtaining approval from the institutional ethics committee. All procedures followed a standardized protocol using four L- or straight miniplates at the pyriform and zygomatic buttress regions bilaterally, without routine bone grafting for gaps of ≤6 mm. Digital lateral cephalograms were obtained preoperatively (T0), immediately postoperatively (T1), and ≥12 months postoperatively (T2). The horizontal position of point A parallel to the Frankfort plane was measured using Dolphin Imaging software. Statistical analyses included independent t-tests and multivariate linear regression.

Results

The mean maxillary advancement at T1 was 5.52±1.18 mm. At a mean follow-up of 14.38±1.29 months, mean horizontal relapse was 1.33±0.31 mm, representing 24.06±2.92% of the surgical advancement. Patients with advancements >5 mm (n=19) showed significantly lower percentage relapse (22.07±2.64% vs. 26.06±3.35%, p=0.003) and less overjet relapse (0.27±0.12 mm vs. 0.41±0.10 mm, p=0.001) than those with ≤5 mm advancement. Multivariate linear regression confirmed that greater magnitude of advancement was an independent negative predictor of percentage relapse (p=0.033). Concomitant mandibular surgery, age, and sex did not significantly affect the stability.

Conclusion

Four-point 2.0-mm titanium miniplate fixation provided reliable long-term skeletal stability after LeFort I maxillary advancement. Larger advancements were more stable than smaller ones, supporting adequate rather than conservative surgical correction. Clinicians can counsel patients that approximately one-quarter of the achieved advancement is typically lost within the first postoperative year with excellent stability thereafter.

## Introduction

LeFort I osteotomy with maxillary advancement is the standard procedure for correcting maxillary retrusion seen in Class III malocclusion, cleft lip and palate, and various syndromic conditions [1]. Rigid fixation using titanium miniplates and screws has largely replaced wire fixation and prolonged maxillomandibular fixation, allowing immediate stability, early return to function, and improved patient comfort [2]. Despite these advances, postoperative relapse remains the most common complication associated with maxillary advancement [3]. Even a small amount of posterior relapse can compromise the achieved facial profile, dental occlusion, and patient satisfaction, sometimes requiring secondary surgery.

Relapse after forward movement of the maxilla is influenced by the extent of advancement, quality of mobilization, soft tissue tension (especially from the palatal mucosa and posterior pharyngeal tissues), direction and magnitude of concomitant mandibular movement, and long-term growth or remodelling in younger patients. This is more commonly observed in patients with cleft lip and palate patients [4]. Although rigid fixation has reduced relapse compared to the era of wire osteosynthesis, relapse rates after maxillary advancement have been reported to be 28.6% with 6.3 mm of maxillary advancement [5]. An accurate assessment of skeletal stability following contemporary LeFort I advancement using rigid fixation is therefore essential to guide surgical planning, predict outcomes, and appropriately counsel patients.

This retrospective study aimed to evaluate the skeletal stability of the maxilla following LeFort I advancement osteotomy stabilized with rigid internal fixation using titanium miniplates and screws. The objectives were to measure the amount of anterior relapse at one-year follow-up, calculate the percentage of relapse relative to the surgical advancement achieved, and identify the influence of the magnitude of advancement (≤5 mm vs. >5 mm), patient age, sex, and concomitant mandibular surgery on relapse.

## Materials and methods

This retrospective clinical study was conducted in the Department of Oral and Maxillofacial Surgery, RKDF Dental College and Research Centre, Bhopal, Madhya Pradesh, India, in May 2023. Ethical clearance was obtained from the Institutional Ethics Committee of the college (RKDF/DC/PG/2023/12E). As the study was purely retrospective and involved only the analysis of existing records and radiographs with no additional patient interaction or intervention, the committee waived the requirement for informed consent.

A priori sample size estimation was performed using the G*Power software (version 3.1.9.2, Heinrich Heine University, Düsseldorf, Germany) [6]. Using a one-sample proportion test, the calculation was based on an anticipated relapse rate of 20%, as reported in the literature [7]. The analysis indicated that 30 patients provided 80% statistical power at an alpha level of 0.05. To account for potential uncertainties and to enhance the robustness of the findings, the final sample size was increased to 32.

Patients were eligible for inclusion if they were aged 18-40 years, had undergone LeFort I maxillary advancement osteotomy (with or without concomitant mandibular surgery) between January 2018 and December 2021 at the department, and had complete standardized digital lateral cephalograms available preoperatively (T0), immediately postoperatively within one week (T1), and at ≥12-month follow-up (T2) time points. Exclusion criteria included patients with incomplete records or radiographs, craniofacial syndromes, prior maxillary or midface surgery, trauma-related deformities, or severe comorbidities (such as uncontrolled diabetes, bleeding disorders) that could affect healing or stability. Patients requiring postoperative maxillomandibular fixation or those with bone gaps >6 mm not grafted with autologous iliac crest bone were also excluded to ensure uniformity in the surgical technique and postoperative protocol.

Surgical procedure

All surgeries were performed under general anesthesia with nasotracheal intubation by the same team of two oral surgeons with more than eight years of clinical experience. A standard maxillary vestibular incision was made from the right first molar region to the left first molar region. Subperiosteal dissection was performed to expose the anterior maxilla, lateral maxillary walls, nasal aperture, and pterygomaxillary regions. A horizontal LeFort I osteotomy was performed using a reciprocating saw 5 mm above the apices of the teeth and extended posteriorly. Pterygomaxillary disjunction was completed using a curved osteotome, and down-fracture was achieved using Tessier mobilizers and Rowe’s disimpaction forceps. Complete mobilization was confirmed by the free forward movement of the maxilla without resistance. Predetermined advancement was achieved using an intermediate acrylic splint. Rigid fixation was achieved using four L-shaped or straight 2.0 mm titanium miniplates with monocortical screws (Synthes Maxillofacial System, Synthes GmbH, Zuchwil, Switzerland), two plates at the pyriform region, and two plates at the zygomatic buttress region bilaterally. Bone gaps greater than 6 mm were grafted with autologous anterior iliac crest corticocancellous blocks. The wound was closed using 3-0 polyglactin 910 sutures. Maxillomandibular fixation was not performed. All patients received the same postoperative protocol: intravenous antibiotics for five days, soft diet for six weeks, and regular follow-up.

Radiographic assessment

Standardized digital lateral cephalograms were obtained using the same machine (Instrumentarium Orthopantomograph® OP300, Instrumentarium Dental, Tuusula, Finland) at three time points: T0, preoperative (within two weeks before surgery); T1, immediate postoperative (within seven days); T2, follow-up for a minimum period of 12 months postoperatively. All cephalograms were traced and measured by a single investigator using Dolphin Imaging Software version 11.95 Premium (Dolphin Imaging & Management Solutions, Chatsworth, CA, USA). Horizontal relapse was measured as the change in the anteroposterior position of Point A parallel to the Frankfort horizontal (FH) plane from T1 to T2.

Calibration and reliability

Fifteen randomly selected cephalograms were retraced after a three-week interval by the primary investigator and independently by a second senior faculty member. Intra-observer and inter-observer reliabilities were assessed using the intraclass correlation coefficient (ICC). The intra-observer ICC was 0.98 (95% CI: 0.96-0.99), and the inter-observer ICC was 0.97 (95% CI: 0.94-0.99). Method error (Dahlberg’s formula) was 0.29 mm for horizontal measurements.

Statistical analysis

Statistical analyses were performed using the Statistical Package for Social Sciences (SPSS) version 22.0 (IBM Corp., Armonk, New York, USA). Categorical variables were presented as frequencies and percentages. Continuous variables, confirmed to be normally distributed via the Shapiro-Wilk test, are presented as the mean±standard deviation. An independent samples t-test was used for the univariate analysis of group differences in relapse. Multivariate linear regression was conducted to identify independent predictors of relapse following Le Fort 1 advancement, with statistical significance set at p<0.05.

## Results

A descriptive analysis of the study population is presented in Table 1. The cohort, with a mean age of 25.13±3.42 years, underwent maxillary advancement with a mean horizontal advancement (T1) of 5.52±1.18 mm. Postoperative follow-up revealed a mean horizontal relapse of 1.33±0.31 mm at T3, representing a percentage relapse of 24.06±2.92% from a mean advancement amount of 5.13±0.95 mm, over an average follow-up duration of 14.38±1.29 months. Cephalometrically, the pre-surgical (T0) overjet was -2.57±0.88 mm, which was corrected to 2.91±0.46 mm post-advancement (T1), with a subsequent minor relapse of 0.35±0.13 mm at T3. The SNA (an angle between SN (Sella-Nasion as SN, where Sella point is the midpoint of sella turcica) plane and NA line (a line from Nasion as N point, which is the most anterior point on frontonasal suture to point A, which is the deepest point in anterior concavity of maxilla)) angle improved by 7.63±1.010 from T0 to T1, with a small mean relapse of 1.49±0.270 at the final follow-up (T2).

**Table 1:** Baseline characteristics and cephalometric measurements of the study cohort. SNA: An angle between SN (Sella-Nasion as SN, where Sella point is the midpoint of sella turcica) plane and NA line (A line from Nasion as N point, which is the most anterior point on frontonasal suture to point A, which is the deepest point in anterior concavity of maxilla), CI: confidence interval, TO: preoperative, TI: immediate postoperative within one week, T2: final follow-up (mean 14.4 months). Values are presented as mean and standard deviation (SD).

Parameters	Minimum	Maximum	95% CI for mean	Mean±SD
Age (years)	19	32	23.89-26.36	25.13±3.42
Horizontal advancement at T1 (mm)	4	8.6	5.09-5.94	5.52±1.18
Horizontal relapse at T2 (mm)	0.8	2	1.21-1.44	1.33±0.31
Percentage horizontal relapse (%)	19.4	30	23.01-25.11	24.06±2.92
Amount of advancement (mm)	3.7	6.9	4.79-5.48	5.13±0.95
Follow-up duration (months)	12	16	13.91-14.84	14.38±1.29
Overjet T0 (mm)	-4.2	-1.4	-2.89 to -2.25	-2.57±0.88
Overjet T1 (mm)	2.4	3.8	2.75-3.08	2.91±0.46
Overjet T2 relapse (mm)	0.2	0.6	0.3-0.4	0.35±0.13
SNA at T0 (degree)	76	87	77.35-79.27	78.31 ± 2.66
SNA at T1-T0 (degree)	6	9	7.26-7.99	7.63±1.01
SNA at T2-T1 (degree)	1	1.9	1.4-1.59	1.49±0.27

Descriptive analysis of the categorical variables is presented in Table 2. The study population consisted of 18 (56.3%) women and 14 (43.8%) men. LeFort I osteotomy alone was the most common surgical procedure performed in 20 (62.5%) patients, while bimaxillary surgery was performed in 12 (37.5%) patients. When categorized according to the amount of mandibular advancement, 18 (56.3%) patients had an advancement of less than 5 mm.

**Table 2:** Distribution of categorical variables. Values are presented as frequency (n) and percentage (%).

Parameters	Variables	Frequency (n)	%
Sex	Female	18	56.3%
	Male	14	43.8%
Type of surgery	LeFort I only	20	62.5%
	Bimaxillary	12	37.5%
Advancement group	≤5 mm	18	56.3%
	>5 mm	14	43.8%

Univariate analysis comparing patients based on the magnitude of advancement (≤5 mm vs. >5 mm) is presented in Table 3. Statistically significant differences were observed in several key outcomes. The group with larger advancements (>5 mm) demonstrated significantly less overjet relapse (0.27±0.12 mm vs. 0.41±0.10 mm; p=0.001) and a lower percentage of horizontal relapse (22.07±2.64% vs. 26.06±3.35%; p=0.003), despite having a significantly greater amount of initial surgical advancement (6.07±0.50 mm vs. 4.40±0.41 mm; p<0.001). The follow-up duration was also significantly shorter in the >5 mm group (13.71±0.91 months vs. 14.89±1.32 months; p=0.008). No significant differences were found between the two groups regarding age, SNA angle relapse, sex distribution, or type of surgery performed (all p>0.05).

**Table 3:** Univariate comparison of skeletal stability according to magnitude of maxillary advancement. SNA: An angle between SN (Sella-Nasion as SN, where Sella point is the midpoint of sella turcica) plane and NA line (A line from Nasion as N point, which is the most anterior point on frontonasal suture to point A, which is the deepest point in anterior concavity of maxilla), TI: Immediate postoperative within one week, T2: Final follow-up (mean 14.4 months). Test value obtained by independent t test (continuous data) and chi-square test (categorical data) between cohorts. *p<0.05 denotes statistical significance.

Parameters	>5 mm	≤5 mm	Test value	p-value	Cohen's d
Age (years)	24.14±3.82	25.89±2.97	-1.46	0.156	0.52
Change in SNA angle (T2-T1) in degrees	1.46±0.27	1.52±0.27	-0.68	0.504	0.24
Overjet at T2 in mm	0.27±0.12	0.41±0.10	-3.55	0.001*	1.26
Amount of advancement (mm)	6.07±0.50	4.40±0.41	10.33	0.001*	3.68
Percentage relapse (%)	22.07±2.64	26.06±3.35	-0.01	0.003*	0.62
Follow-up duration (months)	13.71±0.91	14.89±1.32	-2.83	0.008*	1.01
Sex (Male)	8 (25%)	6 (18.75%)	1.81	0.178	0.33
Female	6 (18.75%)	12 (37.5%)			
Type of surgery (LeFort I)	8 (25%)	12 (37.5%)	0.31	0.581	0.14
Bimaxillary	6 (18.75%)	6 (18.75%)			

Multivariate linear regression analysis was performed to identify significant predictors of relapse following LeFort I surgery, and the results are detailed in Table 4. The analysis revealed that the magnitude of surgical advancement was a significant independent predictor (B=-2.617, p=0.033), indicating that for every 1 mm increase in advancement, the percentage of relapse decreased by approximately 2.62%. Furthermore, the advancement group itself was a strong predictor, with both the ≤5 mm group (B=39.785, p=0.001) and the >5 mm group (B=43.389, p=0.001) showing a significant association with relapse percentage compared to the baseline. A longer follow-up duration was also associated with a slight but significant decrease in relapse (B=-0.422, p=0.039). In contrast, SNA and overjet relapses were not found to be significant independent predictors in this model (p=0.289 and p=0.574, respectively).

**Table 4:** Multivariate linear regression analysis of predictors of percentage horizontal relapse. SNA: An angle between SN (Sella-Nasion as SN, where Sella point is the midpoint of sella turcica) plane and NA line (A line from Nasion  as N point, which is the most anterior point on frontonasal suture to point A, which is the deepest point in anterior concavity of maxilla), CI: confidence interval. *p<0.05 denotes statistical significance.

Model	Coefficient	Test value (t)	p-value	95% CI for lower bound	95% CI for upper bound
Relapse in SNA angle (degrees)	2.146	1.083	0.289	-1.926	6.218
Relapse in overjet (mm)	-2.901	-0.57	0.574	-13.363	7.561
Follow-up duration (months)	-0.422	-0.858	0.039*	-1.432	-0.588
Amount of maxillary advancement (mm)	-2.617	-2.25	0.033*	-5.009	-0.226
Group with ≤ 5 mm of advancement	39.785	3.86	0.001*	18.598	60.972
Group with ≥ 5 mm of advancement	43.389	3.993	0.001*	21.053	65.725

## Discussion

The present retrospective study evaluated skeletal stability following LeFort I maxillary advancement stabilized with four 2.0-mm titanium miniplates and monocortical screws in 32 patients. At a mean follow-up of 14.4 months, the mean horizontal relapse at point A was 1.33±0.31 mm, corresponding to 24.1% of the surgical advancement (mean advancement 5.52 mm). A previous study reported that postoperative changes greater than 2 mm are considered clinically significant relapses [8]. Our study reported a relapse of 1.33 mm. Dowling et al. [9] reported a relapse of >2 mm in 14% of patients and 18% of the total surgical advancement.

An intriguing finding was the inverse relationship between the magnitude of advancement and percentage of relapse. Patients aged >5 mm exhibited a significantly lower percentage of relapse and less overjet relapse, despite receiving larger movements. Multivariate regression confirmed that every additional millimeter of advancement reduced the percentage of relapse by approximately 2.6%. By contrast, Chen et al. [10] reported a significant positive correlation between the amount of advancement and relapse. Similar findings were reported by Fahradyan et al. [5]. The difference in the results could be due to the fact that they conducted their study on patients undergoing two-jaw surgery. Larger advancements create wider bone gaps that promote greater osteogenesis and fibrotic scarring in a more favorable vector, counteracting soft tissue pull [9,11]. In contrast, smaller advancements (≤5 mm) often leave narrower gaps that heal predominantly by fibrous rather than bony union, allowing greater elastic recoil of stretched periosteal-palatal tissues and the posterior pharyngeal envelope [3].

Concomitant mandibular surgery (double-jaw surgery) did not emerge as a significant predictor of relapse in our cohort, corroborating previous studies [12,13]. This contrasts with older reports from the wire-fixation era, where counterclockwise rotation of the maxillomandibular complex dramatically increased maxillary relapse due to the supero-posterior pull of the pterygomasseteric sling. Rigid fixation appears to neutralize this effect, providing sufficient resistance even in bimaxillary cases [14,15]. Age and gender also failed to reach statistical significance within the adult population (mean age 25 years in our sample), and chronological age had minimal influence on stability once rigid fixation was employed [16].

Clinical implications and limitations

The findings of this study have several practical implications in surgical practice and patient counselling. Clinicians can expect approximately 75%-78% long-term skeletal stability at one year when using four-point 2.0-mm titanium miniplate fixation without routine bone grafting for advancements of up to 6-7 mm, making this a reliable and minimally invasive approach in non-cleft patients. The observation that larger advancements (>5 mm) are proportionally more stable than smaller ones challenges the traditional tendency to undercorrect out of fear of relapse and supports deliberate slight overcorrection (1-1.5 mm) in Class III and cleft cases to compensate for the predictable 22%-26% posterior relapse that occurs predominantly in the first six to eight weeks. Concomitant mandibular setback or advancement does not appear to compromise maxillary stability under rigid fixation, allowing surgeons to perform bimaxillary surgery confidently when indicated. Patients can be informed preoperatively that roughly one-quarter of the achieved advancement will be lost in the first year; however, further significant relapse beyond 12-18 months is uncommon.

Despite these strengths, this study has some limitations. The retrospective design introduces potential selection bias and information bias, and the modest sample size of 32 patients, although adequately powered for the primary outcome, restricts detailed subgroup analysis. Reliance on two-dimensional lateral cephalometry may underestimate true three-dimensional relapse, particularly rotational and vertical changes in the zygomatic buttress and pterygoid regions. Follow-up averaged only 14.4 months, minor late relapse beyond three to five years cannot be excluded, especially in younger individuals still exhibiting craniofacial remodelling. Finally, the results apply specifically to four-plate 2.0-mm titanium fixation performed by an experienced team; extrapolation to two-plate techniques, biodegradable systems, or less-standardized protocols should be performed cautiously.

## Conclusions

LeFort I maxillary advancement stabilized with a four-point 2.0-mm titanium miniplate rigid fixation provides predictable and clinically acceptable long-term skeletal stability in both isolated maxillary and bimaxillary procedures. Larger surgical advancements have demonstrated proportionally greater stability than smaller movements, challenging the historical practice of deliberate under-correction in anticipation of excessive relapse. Concomitant mandibular surgery did not compromise maxillary stability when rigid fixation was employed, and neither age nor sex significantly influenced the outcome in adult patients. These findings reinforce the reliability of contemporary rigid fixation techniques, support confident correction of maxillary deficiency without routine bone grafting for moderate advancements, and enable more accurate preoperative counselling regarding the expected postoperative changes. While minor posterior relapse remains inevitable, the majority of patients achieve stable and esthetically satisfactory results with this standardized approach. The findings of this study should be taken cautiously as it was retrospective in nature.
